# Genetic predictive links between oropharyngeal microecology and obstructive sleep apnea syndrome: expanding the perspective of etiology

**DOI:** 10.1080/20002297.2026.2701568

**Published:** 2026-08-18

**Authors:** Libo Zhao, Yang Zhang, Li Lin, Tianyi Wang, Yaodong Ding, Wenjie Wang, Zehao Zhao, Jiayi Han, Lin Liu, Yong Zeng

**Affiliations:** a Department of Cardiology, Beijing Anzhen Hospital, Capital Medical University, Beijing, China; b Department of Respiratory and Critical Care Medicine, the Second Medical Center & National Clinical Research Center for Geriatric Diseases, Chinese PLA General Hospital, Beijing, China

**Keywords:** Obstructive sleep apnea syndrome (OSAS), oropharyngeal microecology, Mendelian randomization (MR), genetic epidemiology, GWAS

## Abstract

**Background:**

Obstructive sleep apnea syndrome (OSAS), a global public health concern, is linked to oropharyngeal microbiota, but observational studies cannot confirm their causal association.

**Objective:**

To explore the potential causal relationship between oropharyngeal microecology and OSAS.

**Methods:**

The data related to the oropharyngeal microbiome and this disease were obtained from China Nucleotide Sequence Archive and genome-wide association studies. Moreover, we screened qualified single nucleotide polymorphisms as instrumental variables. Two-sample Mendelian randomization bidirectional analysis was conducted using methods such as Wald ratio and inverse-variance weighted. The horizontal pleiotropy was judged by the MR-Egger intercept, and the sensitivity analysis was conducted by the “Leave-one-out” method and Cochran's Q assessment.

**Results:**

Interferences in the abundance of specific strains of oropharynx (overall, the Firmicutes/Bacteroidetes phylum ratio ↑) raised the risk of OSAS, such as increased Streptococcus mgs 2253 (OR = 4.280) and decreased Prevotella mgs 3161 (OR = 0.218). Conversely, OSAS also affected the abundance of some oropharyngeal microorganisms, such as a decrease in Neisseria lactamica mgs 343 and an increase in Pauljensenia mgs 2308.

**Conclusion:**

This study emphasizes the causal relationship between oropharyngeal microecology and OSAS, expands the new etiological mechanism, and provides potential novel strategies for the prevention and treatment of OSAS.

## Introduction

Obstructive sleep apnea syndrome (OSAS) affects approximately 1 billion people globally. It primarily arises from the recurrent collapse of the upper airway during sleep, leading to breathing interruptions, intermittent hypoxia, increased sympathetic activity, sleep fragmentation and disruption of physiological homeostasis. Patients with OSAS often experience poor sleep quality, which can result in excessive daytime sleepiness, fatigue and difficulty concentrating [1]. Furthermore, OSAS significantly impacts multiple organ systems and may exacerbate cardiovascular diseases and metabolic dysfunction. Just as the existing research has shown that OSAS is clearly associated with the severity of coronary artery disease and major adverse cardiovascular events [2,3]. Nonetheless, OSAS remains considerably underdiagnosed and undertreated, posing a substantial economic and healthcare burden on society [4].

The etiology of OSAS is multifactorial, involving factors such as alterations in craniofacial anatomy, tonsillar hypertrophy, upper airway edema, reduced muscle tone in airway muscles and obesity [5]. It is evident that the diameter and volume of the upper airway serve as key direct determinants. However, the underlying mechanisms contributing to these direct causes are not yet entirely understood.

In recent years, the human microbiota has emerged as a therapeutic target for preventing and treating chronic diseases, as well as for promoting longevity and health. Microorganisms, which have existed for over 3 billion years, have played a fundamental role in shaping Earth's environment and enabling the evolution of other life forms. Nowadays, microbes represent the most diverse forms of life on the planet, accounting for an estimated 99% of all species. They inhabit nearly every environment, from deep-sea trenches to the human oral and intestinal tracts. Importantly, the human microbiome exerts profound functional influences on the host [6]. By effectively managing microbial health, we may advance strategies for sustainable human health.

Previous studies have revealed that OSAS and high-altitude hypoxia are associated with changes in the oropharyngeal microbiome [7,8]. A study from Colombia also suggested a correlation between periodontitis staging and OSAS. Patients with both OSAS and periodontitis exhibited reduced biodiversity in the oropharyngeal microbiota, and an increase in specific taxa such as *Candida albicans* may exacerbate the severity of periodontal disease in OSAS patients [9]. Similar findings have been reported in gut microbiota research. Wang et al. concluded that OSAS patients show an increased abundance of *Firmicutes* and a reduction in *Bacteroidetes* and *Rikenellaceae* [10]. Additionally, animal experiments conducted by Isabel et al. indicated a higher *Firmicutes*/*Bacteroidetes* ratio in fecal samples from OSAS mouse models, suggesting an imbalance in the intestinal microecology [11]. These correlative findings provide a foundational step toward exploring metabolic influences and mechanistic insights. Clarifying a causal relationship between the microbiome and OSAS is of practical significance for the prevention and management of this prevalent disorder.

However, most existing studies have described only associations between microbial ecology and OSAS without inferring causality. Randomized controlled trials (RCTs) represent the gold standard in evidence-based medicine for establishing causal relationships [12], yet they are often difficult to implement because of ethical constraints, long observation periods and high costs. With the advancement of research methods, Mendelian randomization (MR) has emerged as a robust method in genetic epidemiology that uses genetic variants as instrumental variables (IVs) to assess whether an exposure causally influences an outcome [13]. MR mitigates limitations inherent in observational studies, such as confounding and reverse causation, while circumventing many practical constraints of RCTs, since genetic variants are inherited randomly and naturally. This approach has gained widespread recognition as an innovative strategy in recent epidemiological research. Nevertheless, there are hardly any MR analyses conducted to investigate the potential causal relationship between the oropharyngeal microbiota and the risk of OSAS.

Therefore, this study employs MR analysis to thoroughly explore the causal link between the oropharyngeal microbiota and OSAS and to identify specific pathogenic or protective bacterial taxa. The aim of our research was to provide new genetic evidence and insights into the role of the oropharyngeal microbiome in the etiology and pathogenesis of OSAS.

## Materials and methods

### Design scheme

This study was a bidirectional MR analysis, with the oropharyngeal microbiota as the exposure factor and OSAS as the outcome, to explore the causal relationship between the oropharyngeal microbiota communities and OSAS. Firstly, the selection of instrumental variables (IVs) adhered to the three main principles confirmed previously: (a) the correlation assumption, which indicates that IVs are strongly associated with the exposure factors; (b) the independence assumption, which means that IVs should not correlate with the outcome through confounding factors; and (c) the exclusive assumption, which ensures that IVs influence the outcome solely through the exposure factors without alternative pathways. Following these principles, single nucleotide polymorphisms (SNPs) were rigorously screened as IVs from each dataset in the bidirectional analysis. The threshold for identifying relevant SNPs was set at *p* < 1 × 10^−5^ were retained to ensure the robustness of the IVs, which were closely related to the phenotype. Moreover, ensured that the SNPs were independent of each other and filtered out data related to linkage disequilibrium (r^2^ = 0.001, genetic distance = 10,000). Calculated and examined the F-statistic size of each SNPs. To eliminate the influence of weak instrumental variables, retained the SNPs corresponding to F > 10 to ensure the robustness of IVs. Meanwhile, potential confounding factors such as smoking, drinking and obesity, which might affect the forward and reverse exposures in the database (*p* < 5 × 10^−5^), were removed. Since this was a bidirectional analysis, the selection method of IVs during the reverse analysis was the same as above.

### Data sources

This study applied the oropharyngeal microbiota communities of data was from the China Nucleotide Sequence Archive (CNSA) (https://ftp.cngb.org/pub/CNSA/data5/CNP0001664/), and the corresponding gene sequencing data were derived from 2017 swab samples taken from the dorsal surface of the tongue and the oropharynx for 1915 individuals in China. The OSAS corresponding SNPs data were derived from the ebi-a-GCST90018696 dataset in the genome-wide association studies (GWAS) [14], which included 473 cases and 177,864 controls, and the total number of SNPS was 12,454,637. Considering the consistency with the population for oropharyngeal microbial collection, the research subjects in both datasets were from East Asia, without gender restrictions.

### Statistical processing

Overall, statistical analysis was conducted using R 4.2.1 two-sample MR software, and it was considered that the results had statistical significance when *p* < 0.05. In the analysis, the results of the inverse variance weighted (IVW) method and the Wald ratio method were mainly used, and the analytical methods such as MR-Egger were adopted as supplementary methods. Furthermore, the calculation of a single SNP effect was analyzed using the Wald ratio method [15]. The association results were expressed as odds ratios (OR) and the corresponding 95% confidence intervals (CI). The MR-Egger intercept term was analyzed to determine horizontal pleiotropy. If the Egger-intercept was infinitely close to 0 or the *p*-value was greater than 0.05 with no statistical significance, it was declared that there was no gene pleiotropy. The Cochran's Q statistic was used to evaluate heterogeneity. If *p* > 0.05, it indicated the absence of heterogeneity. Funnel plots were drawn to observe the distribution of SNPs, and the reliability of MR analysis was evaluated by symmetry. Sensitivity analysis was conducted using the ‘leave-one-out’ method (that was, removing individual SNP seriatim and analyzing the combined effect of the remaining SNPs) to evaluate one by one whether a single SNP had an impact on the association between exposure factors and outcomes. In order to avoid false positivity in the association results due to multiple tests, false discovery rate (FDR) correction was performed, and *P*_FDR_ < 0.1 was considered statistically significant [16]. When *p* < 0.05 and *P*_FDR_ ≥ 0.1, it was considered as potential associations between the oropharyngeal microbiota and OSAS.

## Results

### Oropharyngeal microbiota in MR analysis

A total of 1082 oropharyngeal microbial taxa were included in this study, spanning 10 phyla, 15 classes, 30 orders, 52 families and 199 genera. After screening based on the above criteria, 5384 SNPs associated with the oropharyngeal microbiota were identified. All F-statistics exceeded 10, thereby eliminating bias related to weak instrumental variables.

### Forward associations: oropharyngeal microbiota on OSAS

The association analysis between oropharyngeal microbiota and OSAS was primarily conducted using the inverse-variance weighted (IVW) and Wald ratio methods. The results revealed the following:

Within the *Firmicutes* phylum, *Streptococcus* mgs 2821 (OR = 0.342, *p* = 0.007), *Streptococcus* mgs 988 (OR = 0.226, *p* = 0.032) and *Gemella* mgs 2318 (OR = 0.419, *p* = 0.018) were negatively associated with OSAS risk. In contrast, *Granulicatella* mgs 1782 (OR = 5.306, *p* = 0.010), *Streptococcus* mgs 1535 (OR = 2.895, *p* = 0.020), *Streptococcus* mgs 2253 (OR = 4.280, *p* = 0.046), *Solobacterium* mgs 2820 (OR = 3.817, *p* = 0.023), *Solobacterium* mgs 2241 (OR = 3.370, *p* = 0.035) and *Centipeda infelix* mgs 582 (OR = 4.733, *p* = 0.028) were positively associated with OSAS risk.

In the *Bacteroidetes* phylum, *Prevotella multisaccharivorax* mgs 669 (OR = 0.373, *p* = 0.010), *Prevotella* mgs 3161 (OR = 0.218, *p* = 0.018), *Prevotella* mgs 1437 (OR = 0.226, *p* = 0.021), *Weeksellaceae* unclassified mgs 3040 (OR = 0.234, *p* = 0.025) and *Weeksellaceae* unclassified mgs 791 (OR = 0.253, *p* = 0.025) were negatively associated with OSAS. Conversely, *Prevotella denticola* mgs 3531 (OR = 5.077, *p* = 0.027) may increase the risk of OSAS.

In the *Proteobacteria* phylum, an increased abundance of *Haemophilus* mgs 1265 (OR = 4.837, *p* = 0.036) may elevate OSAS risk. Several species of *Saccharimonadaceae* and *Neisseriaceae*, as well as *Kingella kingae* mgs 3078, were associated with a reduced risk of OSAS (all of OR < 1.000, *p* < 0.05), as shown in Table 1. Within the *Spirochaetota* phylum, both *Treponema* C mgs 1992 and *Treponema* A (at the genus level) may represent risk factors for OSAS. In the *Fusobacteriota* phylum, *Streptobacillus* mgs 588 and *Fusobacterium nucleatum* mgs 3334 may serve as protective factors against OSAS. Among the *Actinobacteriota* phylum, all three species listed in Table 1 were associated with an increased risk of OSAS.

**Table 1.** MR analysis results of the forward associations between oropharyngeal microbiota and OSAS.

	β	SE	Number of SNPs	MR method	OR (95% CI)	**p*-*value	*P*_FDR_
***Firmicutes***							
*Streptococcus* mgs 2821	−1.074	0.396	3	IVW	0.342 (0.157–0.742)	0.007	0.99
*Streptococcus* mgs 988	−1.486	0.694	1	Wald ratio	0.226 (0.058–0.882)	0.032	0.86
*Granulicatella* mgs 1782	1.669	0.649	1	Wald ratio	5.306 (1.488–18.922)	0.010	0.86
*Streptococcus* mgs 1535	1.063	0.458	2	IVW	2.895 (1.180–7.102)	0.020	0.99
*Streptococcus* mgs 2253	1.455	0.729	1	Wald ratio	4.280 (1.025–17.868)	0.046	0.98
*Solobacterium* mgs 2820	1.339	0.589	1	Wald ratio	3.817 (1.203–12.105)	0.023	0.86
*Solobacterium* mgs 2241	1.215	0.578	1	Wald ratio	3.370 (1.086–10.463)	0.035	0.86
*Centipeda infelix* mgs 582	1.555	0.707	1	Wald ratio	4.733 (1.185–18.907)	0.028	0.86
*Gemella* mgs 2318	−0.870	0.368	3	IVW	0.419 (0.204–0.862)	0.018	0.99
***Bacteroidetes***							
*Prevotella multisaccharivorax* mgs 669	−0.986	0.382	2	IVW	0.373 (0.177–0.789)	0.010	0.99
*Prevotella* mgs 3161	−1.523	0.643	1	Wald ratio	0.218 (0.062–0.770)	0.018	0.86
*Prevotella* mgs 1437	−1.487	0.642	1	Wald ratio	0.226 (0.064–0.795)	0.021	0.86
*Prevotella denticola* mgs 3531	1.625	0.736	1	Wald ratio	5.077 (1.199–21.481)	0.027	0.86
*Weeksellaceae* unclassified mgs 3040	−1.452	0.649	1	Wald ratio	0.234 (0.066–0.835)	0.025	0.86
*Weeksellaceae* unclassified mgs 791	−1.372	0.613	1	Wald ratio	0.253 (0.076–0.843)	0.025	0.86
***Proteobacteria***							
*Haemophilus* mgs 1265	1.576	0.751	1	Wald ratio	4.837 (1.109–21.087)	0.036	0.86
*Saccharimonadaceae* mgs 1187	−1.874	0.709	1	Wald ratio	0.154 (0.038–0.616)	0.008	0.86
*Saccharimonadaceae* TM7 mgs 385	−1.996	0.686	1	Wald ratio	0.136 (0.035–0.522)	0.004	0.86
*Saccharimonadaceae* TM7 mgs 2077	−1.062	0.467	2	IVW	0.346 (0.138–0.864)	0.023	0.99
*Saccharimonadaceae* TM7 mgs 2917	−0.916	0.449	2	IVW	0.400 (0.166–0.965)	0.041	0.99
*Saccharimonadaceae* TM7 mgs 3169	−0.884	0.449	2	IVW	0.413 (0.171–0.995)	0.049	0.99
*Neisseria sicca* mgs 986	−1.159	0.443	2	IVW	0.314 (0.132–0.748)	0.009	0.99
*Neisseria sicca* mgs 3572	−1.401	0.626	1	Wald ratio	0.246 (0.072–0.840)	0.025	0.86
*Neisseria lactamica* mgs 343	−1.452	0.582	1	Wald ratio	0.234 (0.075–0.732)	0.012	0.86
*Kingella kingae* mgs 3078	−1.109	0.500	2	IVW	0.330 (0.124–0.879)	0.027	0.99
*Haemophilus* mgs 2176	−1.089	0.452	2	IVW	0.336 (0.139–0.815)	0.016	0.99
*Aggregatibacter* mgs 1474	−0.998	0.455	2	IVW	0.369 (0.151–0.899)	0.028	0.99
***Spirochaetota***							
*Treponema* C mgs 1992	1.731	0.645	1	Wald ratio	5.644 (1.595–19.968)	0.007	0.86
*Treponema* A (genus)	1.844	0.699	1	Wald ratio	6.324 (1.604–24.934)	0.008	0.86
***F* *usobacteriota***							
*Fusobacterium* mgs 1937	−1.118	0.428	2	IVW	0.327 (0.141–0.757)	0.009	0.99
*Fusobacterium* mgs 2195	1.064	0.418	3	IVW	2.898 (1.276–6.580)	0.011	0.99
*Streptobacillus* mgs 588	−0.886	0.386	3	IVW	0.412 (0.194–0.879)	0.022	0.99
*Fusobacterium nucleatum* mgs 3334	−1.373	0.635	1	Wald ratio	0.253 (0.073–0.879)	0.030	0.86
***Actinobacteriota***							
*Rothia mucilaginosa* mgs 3219	1.167	0.468	2	IVW	3.210 (1.283–8.034)	0.013	0.99
*Rothia mucilaginosa* mgs 2967	1.270	0.637	1	Wald ratio	3.559 (1.021–12.411)	0.046	0.98
*Pauljensenia* mgs 2308	1.442	0.724	1	Wald ratio	4.230 (1.024–17.470)	0.046	0.98

SE: standard error; MR: Mendelian randomization; OR: odds ratio.

**Figure 1.**
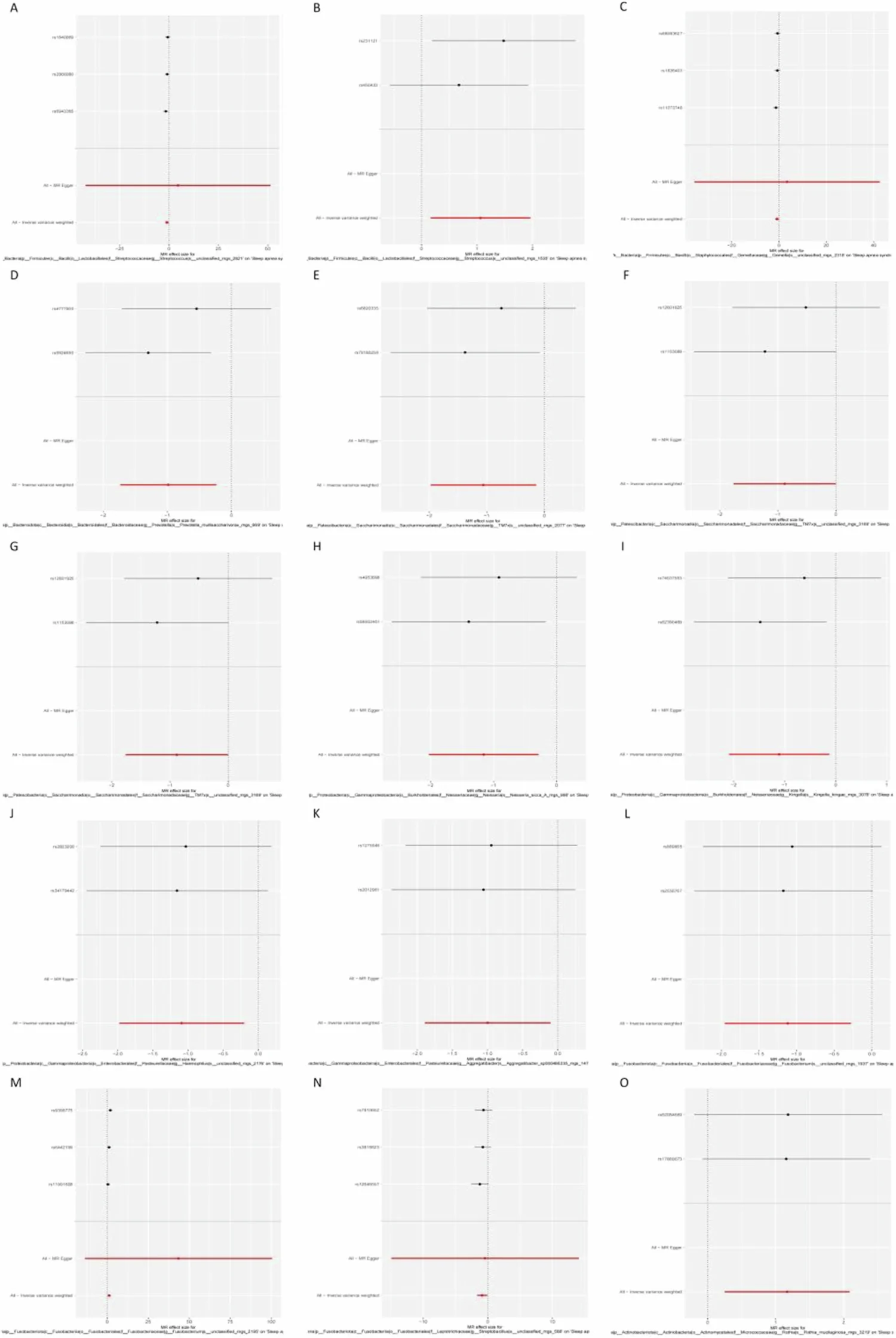
Forest plots of the forward associations from MR analysis results. (a) *Streptococcus* mgs 2821, (b) *Streptococcus* mgs 1535, (c) *Gemella* mgs 2318, (d) *Prevotella multisaccharivorax* mgs 669, (e) *Saccharimonadaceae* TM7 mgs 2077, (f) *Saccharimonadaceae* TM7 mgs 2917, (g) *Saccharimonadaceae* TM7 mgs 3169, (h) *Neisseria sicca* mgs 986, (i) *Kingella kingae* mgs 3078, (j) *Haemophilus mgs* 2176, (k) *Aggregatibacter* mgs 1474, (l) *Fusobacterium* mgs 1937, (m) *Fusobacterium* mgs 2195, (n) *Streptobacillus* mgs 588, (o) *Rothia mucilaginosa* mgs 3219.

Forest plots (Figure 1) illustrated statistically significant associations for the strains with ≥2 SNPs. Overall, an increase in the abundance of most *Firmicutes* species appeared to elevate OSAS risk, while the most significant *Bacteroidetes* species were associated with reduced OSAS risk. Although all the obtained associations had *p*-values < 0.05, false discovery rate (FDR) correction yielded that *P*_FDR_ > 0.1, suggesting that there were some potential associations between the above-mentioned oropharyngeal microbiota and OSAS.

**Figure 2.**
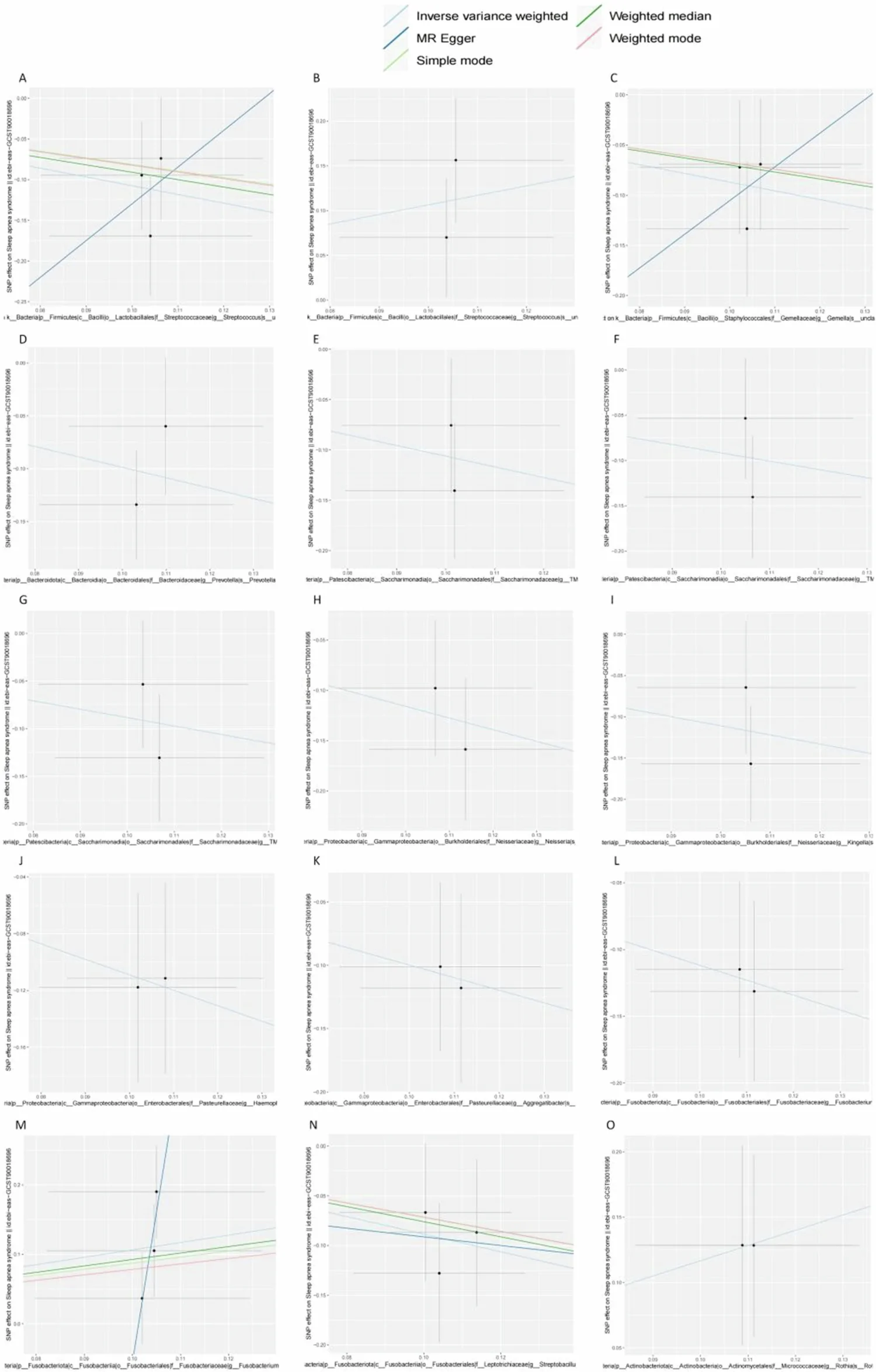
Scatter plots of the forward associations from MR analysis results. (a) *Streptococcus* mgs 2821, (b) *Streptococcus* mgs 1535, (c) *Gemella* mgs 2318, (d) *Prevotella multisaccharivorax* mgs 669, (e) *Saccharimonadaceae* TM7 mgs 2077, (f) *Saccharimonadaceae* TM7 mgs 2917, (g) *Saccharimonadaceae* TM7 mgs 3169, (h) *Neisseria sicca* mgs 986, (i) *Kingella kingae* mgs 3078, (j) *Haemophilus* mgs 2176, (k) *Aggregatibacter* mgs 1474, (l) *Fusobacterium* mgs 1937, (m) *Fusobacterium* mgs 2195, (n) *Streptobacillus* mgs 588, (o) *Rothia mucilaginosa* mgs 3219.

Scatter plots (Figure 2) visualized the causal effects between the oropharyngeal microbiota and OSAS in the MR analysis. The distribution of points in each coordinate system was concentrated, and the slopes of regression lines from different methods were generally consistent for the strains with ≥2 SNPs, particularly when interpreted using the IVW method, supporting a causal relationship between exposure and outcome. An upward slope indicated a positive association, while a downward slope indicated a negative association. However, for *Streptococcus* mgs 2821 (β_MR-Egger_ = 4.515, β_IVW_ = −1.074) and *Gemella* mgs 2318 (β_MR-Egger_ = 3.363, β_IVW_ = −0.870), the comprehensive effect values β directions obtained through MR-Egger and IVW analyses were inconsistent, suggesting instability in the causal relationship. Therefore, these two bacteria were excluded from subsequent discussions.

### Sensitivity analysis of forward associations

**Figure 3.**
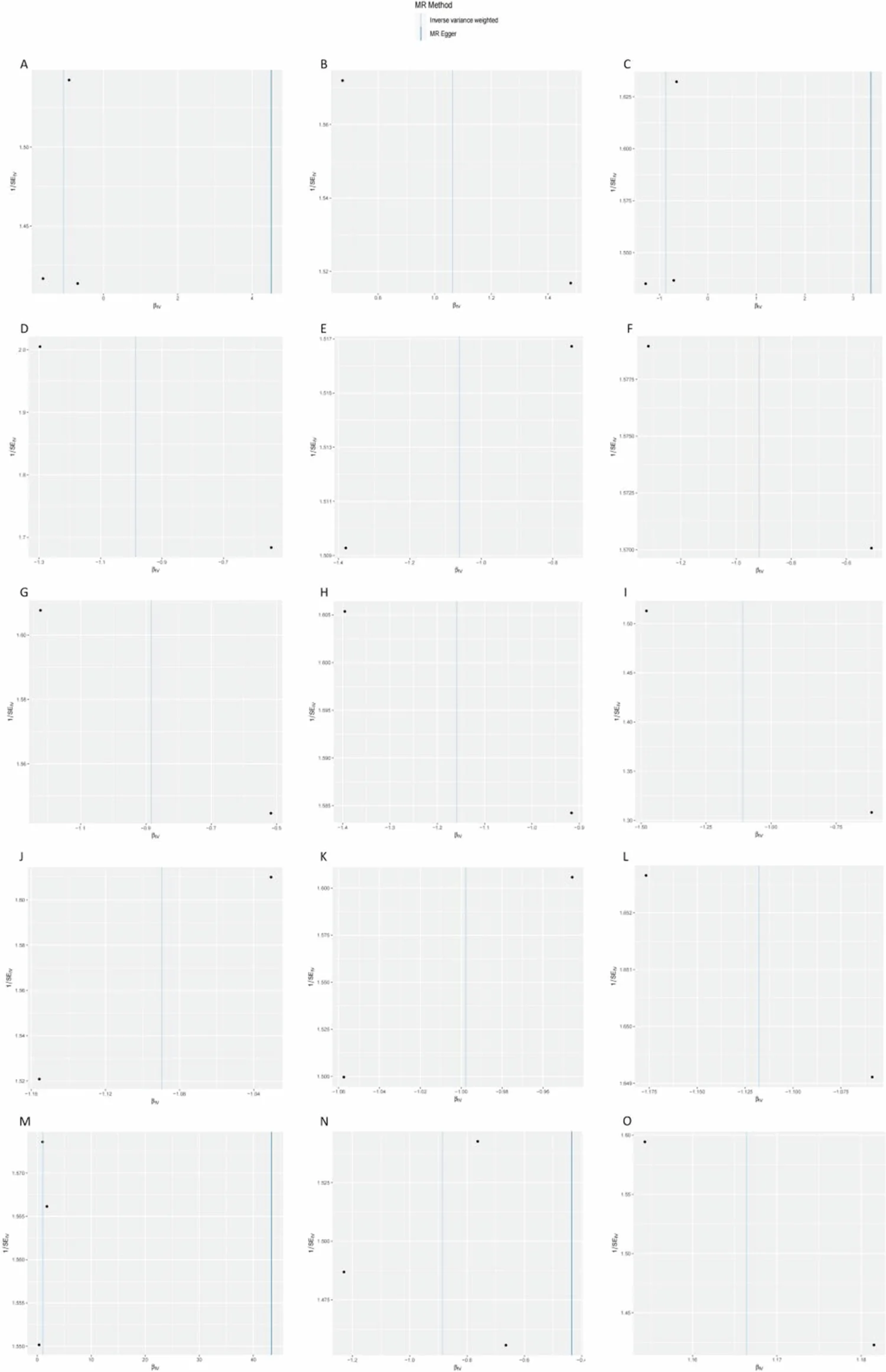
Funnel plots of the forward associations from MR analysis results. (a) *Streptococcus* mgs 2821, (b) *Streptococcus* mgs 1535, (c) *Gemella* mgs 2318, (d) *Prevotella multisaccharivorax* mgs 669, (e) *Saccharimonadaceae* TM7 mgs 2077, (f) *Saccharimonadaceae* TM7 mgs 2917, (g) *Saccharimonadaceae* TM7 mgs 3169, (h) *Neisseria sicca* mgs 986, (i) *Kingella kingae* mgs 3078, (j) *Haemophilus* mgs 2176, (k) *Aggregatibacter* mgs 1474, (l) *Fusobacterium* mgs 1937, (m) *Fusobacterium* mgs 2195, (n) *Streptobacillus* mgs 588, (o) *Rothia mucilaginosa* mgs 3219.

**Figure 4.**
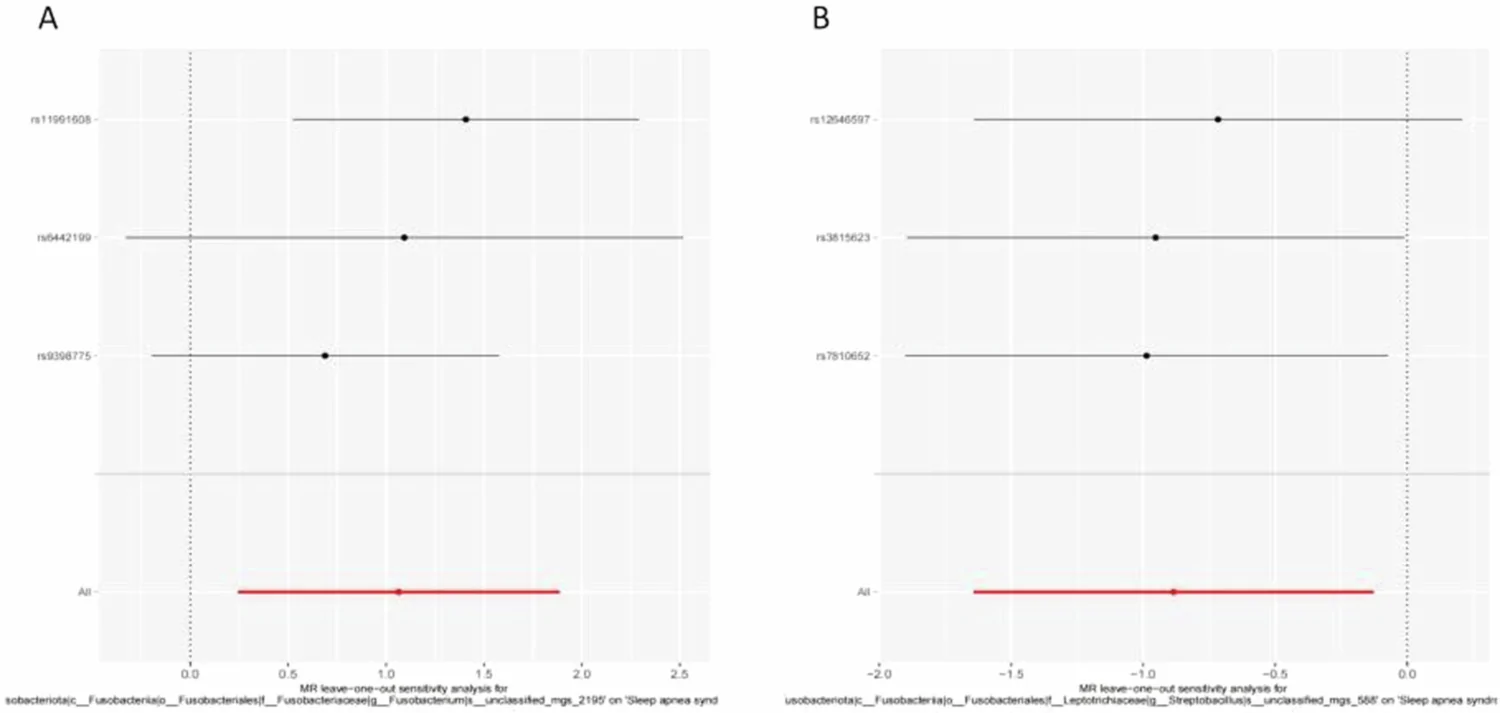
‘Leave-one-out’ analysis of the influence of bacterial strains with multiple SNPs numbers on OSAS. (a) *Fusobacterium* mgs 2195, (b) *Streptobacillus* mgs 588.

(1) Heterogeneity of instrumental variables with statistical significance was assessed using the IVW method. The following strains showed *Q*_IVW_ values with *p* > 0.05, indicating no significant heterogeneity: *Streptococcus* mgs 2821 (*Q*_IVW_ = 0.953, *p* = 0.621), *Streptococcus* mgs 1535 (*Q*_IVW_ = 0.774, *p* = 0.379), *Gemella* mgs 2318 (*Q*_IVW_ = 0.603, *p* = 0.740), *Prevotella multisaccharivorax* mgs 669 (*Q*_IVW_ = 0.943, *p* = 0.331), *Saccharimonadaceae* unclassified mgs 2077 (*Q*_IVW_ = 0.457, *p* = 0.499), *Saccharimonadaceae unclassified* mgs 2917 (*Q*_IVW_ = 0.812, *p* = 0.368), *Saccharimonadaceae* unclassified mgs 3169 (*Q*_IVW_ = 0.617, *p* = 0.432), *Neisseria sicca* mgs 986 (*Q*_IVW_ = 0.292, *p* = 0.589), *Kingella kingae* mgs 3078 (*Q*_IVW_ = 0.734, *p* = 0.392), *Haemophilus* mgs 2176 (*Q*_IVW_ = 0.019, *p* = 0.890), *Aggregatibacter* mgs 1474 (*Q*_IVW_ = 0.015, *p* = 0.902), *Fusobacterium* mgs 1937 (*Q*_IVW_ = 0.019, *p* = 0.890), *Fusobacterium* mgs 2195 (*Q*_IVW_ = 2.568, *p* = 0.277), *Streptobacillus* mgs 588 (*Q*_IVW_ = 0.401, *p* = 0.808) and *Rothia mucilaginosa* mgs 3219 (*Q*_IVW_ = 0.001, *p* = 0.977). (2) The Egger-intercept test was applied to examine the horizontal pleiotropy of non-single SNP instrumental variables. The results showed that the corresponding intercept sizes of the tested bacterial species all approached zero, and all *p* > 0.05. For example, *Streptococcus* mgs 2821 (intercept = −0.581, *p* = 0.853), *Streptobacillus* mgs 588 (intercept = −0.048, *p* = 0.961). The instrumental variables indicating exposure factors met the requirements, and the MR analysis results were not influenced by horizontal pleiotropy. (3) Funnel plots (Figure 3) for the strains with ≥2 SNPs showed symmetrical distribution of SNPs, supporting the relative robustness of the analysis results. (4) Leave-one-out analysis was performed for strains with ≥3 SNPs. The overall causal estimates remained consistent after sequentially excluding each SNP, indicating that the results were not driven or influenced by any individual SNP. So the causal effects remained stable. As shown in Figure 4, the effects of *Fusobacterium* mgs 2195, *Streptobacillus* mgs 588 and other species on OSAS did not change due to the variation in a single SNP.

### Reverse associations: OSAS on oropharyngeal microbiota

**Figure 5.**
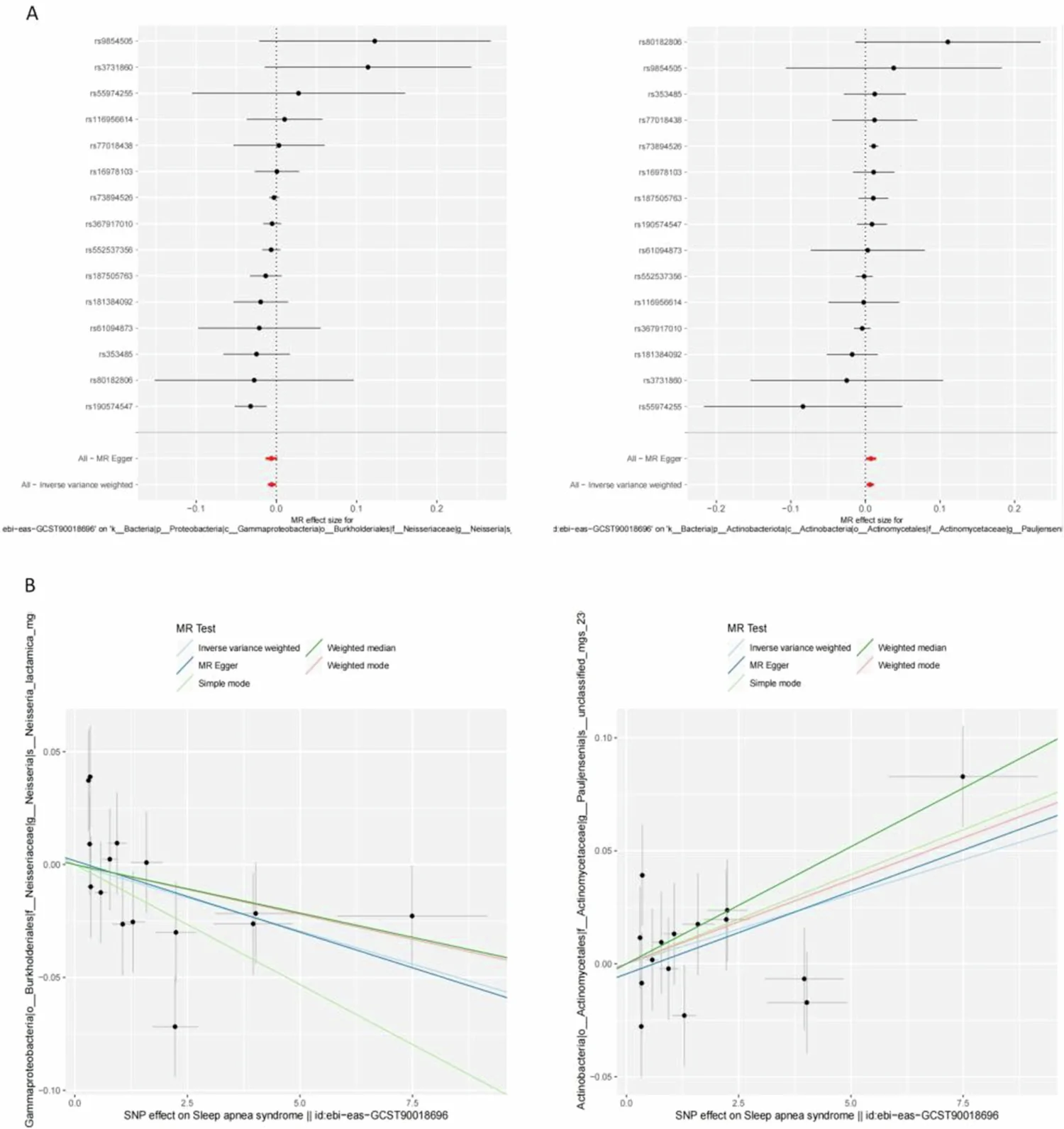
(a) Forest plots and (b) scatter plots of the reverse associations from the MR analysis results. (Left: *Neisseria lactamica* mgs 343; Right: *Pauljensenia* mgs 2308).

When OSAS was set as the exposure factor affecting the aforementioned oropharyngeal microbiota, most reverse associations were not statistically significant (all *p* > 0.05) for the strains with forward associations. However, OSAS was negatively associated with the abundance of *N. lactamica* mgs 343 (OR = 0.994, 95% CI: 0.989–0.999) and positively associated with *Pauljensenia* mgs 2308 (OR = 1.006, 95% CI: 1.002–1.011), both with *p* < 0.05 (Table 2). As shown in the forest plot (Figure 5A), the overall estimate (red line) did not cross the null line, and the entire results were on the unidirectional side of the invalid zero point. Scatter plots (Figure 5B) further demonstrated consistent slope directions across methods, supporting the reverse causal effects of OSAS on these two strains.

**Table 2.** MR analysis results of the reverse associations between OSAS and oropharyngeal microbiota.

	β	SE	Number of SNPs	MR method	OR (95% CI)	*p-*value	*P*_FDR_
**Proteobacteria**							
*Neisseria lactamica* mgs 343	−0.006	0.002	15	IVW	0.994 (0.989–0.999)	0.016	0.36
**Actinobacteriota**							
*Pauljensenia* mgs 2308	0.006	0.002	15	IVW	1.006 (1.002–1.011)	0.007	0.34

SE: standard error; MR: Mendelian randomization; OR: odds ratio.

### Sensitivity analysis of reverse associations

**Figure 6.**
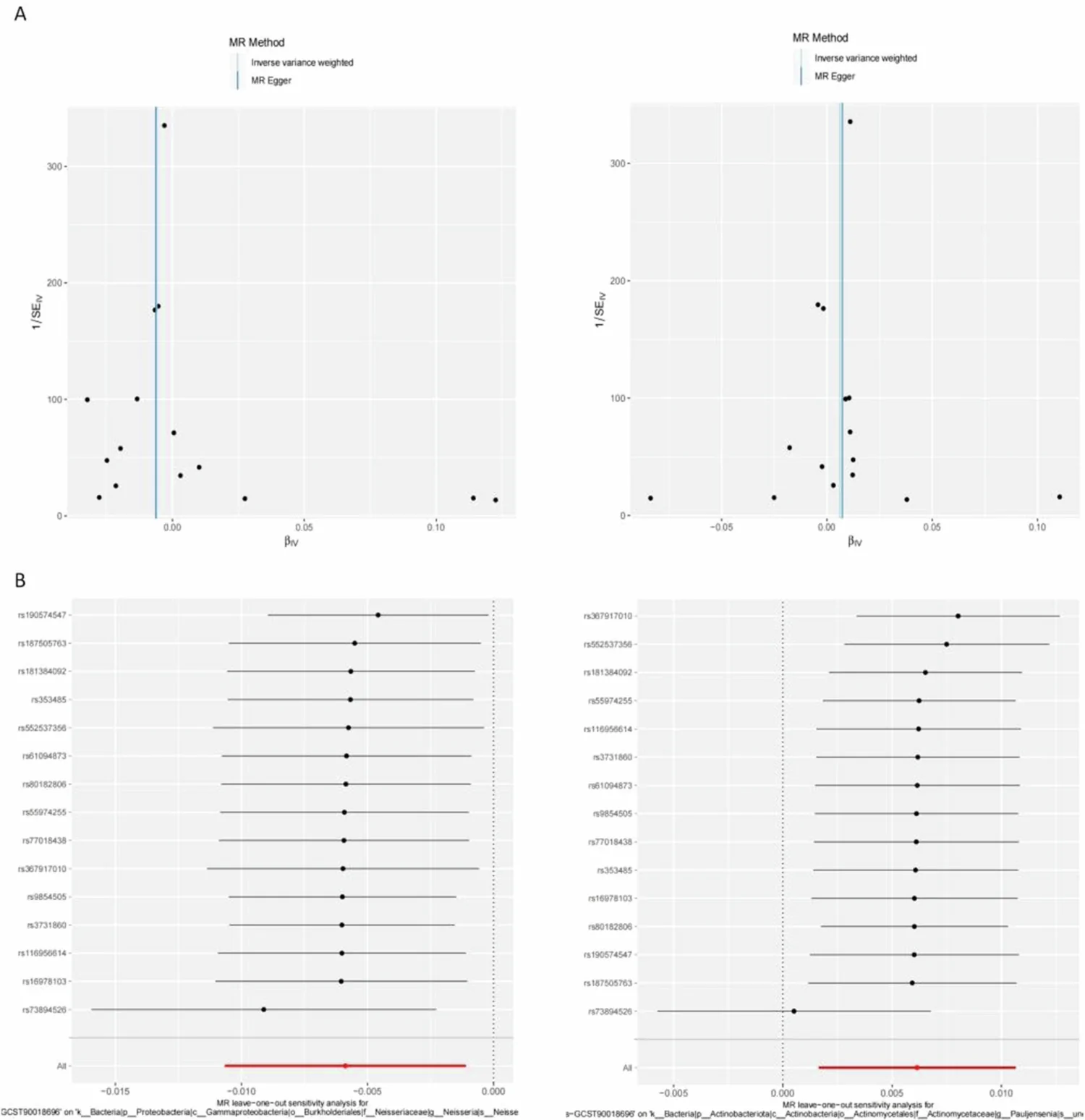
(a) Funnel plots of the reverse associations from the MR analysis results. (b) ‘Leave-one-out’ plots showing the effects of OSAS on two specific bacterial species. (Left: *Neisseria lactamica* mgs 343; Right: *Pauljensenia* mgs 2308).

Cochran's Q test using the IVW method showed no significant heterogeneity for *N. lactamica* mgs 343 (*Q* = 17.533, *p* = 0.229) or *Pauljensenia* mgs 2308 (*Q* = 15.569, *p* = 0.340). The Egger-intercept test revealed no horizontal pleiotropy for either taxon (intercept = 0.002, *p* = 0.861 for mgs 343; intercept = −0.004, *p* = 0.614 for mgs 2308). The corresponding SNPs of OSAS shown in the funnel plots of Figure 6A had good left‒right symmetry, suggesting that the results were relatively stable. Leave-one-out analysis (Figure 6B) indicated that the exclusion of rs73894526 might affect the persistence of the OSAS–*Pauljensenia* mgs 2308 association, but the overall estimate remained on one side of the null line, supporting the robustness and reliability of the results.

## Discussion

Microbiome research has underscored the importance of microbial ecosystems in human health and disease pathogenesis. The vast and diverse microbial communities inhabiting various body sites co-evolve with the host and exert systemic effects, positioning the microbiome as a promising target for disease management. The oropharynx, a critical gateway between the external environment and the body, harbors both commensals and potential pathobionts. Its diverse microbial composition is influenced by local conditions such as temperature, humidity and pH level. Exposure to airborne microbes, pollutants and allergens further shapes this community. Compared to the lower respiratory tract, the upper airway exhibits greater microbial abundance, and the relative ease of sampling makes the oropharyngeal microbiome an active area of investigation.

The oropharyngeal microbiome influences health through multiple mechanisms. Locally, bacteria and their metabolites can penetrate the mucosal barrier and enter the bloodstream—especially given the highly vascularized sublingual area, which is exploited for rapid drug delivery. Some bacteria may invade cranial nerves and reach brain tissue, affecting neural function [17]. Bacterial toxins such as lipopolysaccharide (LPS) promote inflammatory cytokine release, sustaining low-grade inflammation and activating coagulation cascades, thereby compromising cardiovascular health [18]. Inflammatory mediators, including nitric oxide and prostaglandins, contribute to soft tissue edema and reduce upper airway muscle tone. For instance, nitric oxide impairs muscle contractility via the soluble guanylate cyclase-protein kinase G (sGC-PKG) pathway, potentially reducing airway patency. Inflammatory stimulation alters the polarization state of macrophages. Macrophages residing in the neuromuscular circuit can influence and regulate neural activity and muscle contraction by releasing glutamic acid [19]. Oxidative stress, which is closely related to inflammation and promotes each other, can reduce and consume the body's antioxidant capacity. However, maintaining a high level of reducing capacity in the body (represented by nicotinamide adenine dinucleotide) supports muscle strength and function [20]. Moreover, the oropharynx and gut represent the body's largest microbial reservoirs, which are interconnected via the oral‒gut microbiome axis [21]. Oropharyngeal microbes can translocate to the gut via hematogenous routes or through saliva swallowing. Under the mutual influence and joint effect between these two planting locations of microorganisms, they can regulate the host's immunity or induce diseases. For example, research from Xiyuan Hospital, China Academy of Chinese Medical Sciences, found that elevated *Fusobacterium nucleatum* in the oropharynx correlates with oral-gut dysbiosis characterized by increased *Lactobacillus* co-colonization, which is associated with the aggravation of myocardial ischemia-reperfusion injury in diabetes [22]. A study involving 3,570 individuals from Sweden found that OSAS-related hypoxia is associated with lower species diversity in the gut and it could upregulate the gene abundance of metabolic pathways in the microbiome, including the production of propionate from lactic acid [23]. Meanwhile, a large amount of clinical data has indicated that laryngopharyngeal reflux (LPR) is more prevalent in patients with OSAS. Acid reflux can significantly alter the microenvironment of the oropharynx and larynx, affecting pH values, mucosal integrity, local immunity and microbial composition. Moreover, long-term high carbohydrate intake may lead to weight gain, and weight gain is one of the risk factors for OSAS. Obesity may be accompanied by increased intra-abdominal pressure, which increases the chance of reflux. Researchers explained and analyzed that *Streptococcaceae* obtain energy through carbohydrate glycolysis and have strong acid resistance. In addition, long-term excessive intake of carbohydrates in the diet can cause dysfunction of the oropharyngeal environment. In this environment, more organic acids are produced, such as the fermentation byproduct lactic acid, the abundance of *Streptococcaceae* and other acid-resistant bacteria increases, while acid-labile symbiotic bacteria decrease, ultimately leading to disruption of the balance of the oropharyngeal microbiome [24]. Previous clinical studies have also suggested that the pH value of the oropharynx in OSAS patients is lower [25], which is consistent with the increase in the abundance of *Streptococcaceae* as a change in the oropharyngeal environment. Thus, oropharyngeal ecological imbalance may be clearly associated with systemic dysfunction.

Traditional pathophysiological mechanisms of OSAS include anatomical narrowing of the upper airway, abnormal arousal thresholds, impaired pharyngeal dilator responsiveness and unstable ventilatory control, etc [26]. The latter involves complex interactions among peripheral and central chemoreceptors, respiratory center sensitivity, cortical input and gas exchange. Microbial invasion of neural tissues may influence respiratory control, while local inflammation, edema and impaired muscle function may further contribute to OSAS development. Prior studies noted that colonization by *Streptococcus pneumoniae*, *Haemophilus influenzae* and *Staphylococcus aureus* is linked to wheezing, asthma and chronic rhinosinusitis [27], suggesting that microbiota-mediated immune modulation may affect airway homeostasis and OSAS risk. The microbiota in the body is involved in regulating the local and systemic immune responses of the host, causing edema in respiratory system tissues such as the airways, or directly affecting the neural regulation related to respiratory movement, all of which may be related to the occurrence and development of OSAS.

In the current study, several microbial taxa were associated with OSAS risk. Within *Firmicutes*, certain *Streptococcaceae* release virulence factors such as M proteins that trigger inflammation. However, *Streptococcus salivarius*, one of the main bacteria on the oropharyngeal mucosa, may be beneficial, producing fructosidases that inhibit pathogens [28], explaining the mixed protective/harmful effects observed. Increased *Solobacterium* abundance correlates with colorectal cancer via the NF-κB-mediated pro-inflammatory infiltration of myeloid cell subsets [29] and is also linked to halitosis [30]. Gentipedas ferments sugars to acidify the environment, often proliferating in periodontal pockets in patients with periodontitis. In *Bacteroidetes*, *Prevotella* species aid polysaccharide digestion and produce beneficial metabolites like short chain fatty acids (SCFAs) under homeostasis, but may become pathogenic under dysbiotic conditions, contributing to periodontitis and rheumatoid arthritis via arachidonic acid pathways [31]. Researchers at Beijing University of Chinese Medicine discovered that Weeksellaceae reduction following herbal inhalation therapy was correlated with reduced leukocyte infiltration and improved microbial richness [32]. In *Proteobacteria*, multi-omics analyses revealed that a reduction in the abundance of *Saccharimonadaceae* could exacerbate intestinal fibrosis [33], and pathogenic *Haemophilus* species induce steroid-resistant airway inflammation via IL-1β and IL-8 upregulation, leading to a decline in lung function [34].

There are some reports of *Neisseria* causing infective endocarditis [35], most of which are patients with weakened immunity who have undergone thoracotomy aortic valve replacement and hemodialysis. This is not quite consistent with the negative correlation between *Neisseria* abundance and OSAS risk suggested by this study. However, Shen et al. found that *Neisseria sicca* was relatively abundant in healthy individuals without periodontitis. This bacterium can activate inflammasome-mediated pyroptosis by increasing the expression levels of nucleotide-binding oligomerization domain-like receptor protein 3 and the apoptosis-related speck-like protein Caspase-1. The mRNA levels of NF-κB and IL-6 can be down-regulated to inhibit the inflammatory response [36], so its negative association with OSAS risk in our analysis can be supported from the aspect of inflammation control. *Kingella*, fastidious Gram-negative commensals, can translocate across epithelia to cause disseminated infections [37]. The specific mgs 3078 strain appeared protective in this study, possibly via competitive exclusion of pathogens, considering its strict requirements for growth conditions. *Treponema* (belongs to the *Spirochaetes* phylum) was identified as a risk factor; its outer membrane proteins, as virulence factors, modulate neutrophil chemotaxis and it is enriched in the subgingival microbiota of individuals with periodontitis [38]. *Fusobacterium* is an anaerobic bacterium that usually resides in the oropharynx and gastrointestinal tract. It has been widely accepted that it can cause inflammatory stimulation and induce the production of ROS [39]. In *Fusobacteria*, certain species (e.g. mgs 2195) were risk factors, although others showed inverse associations, possibly reflecting genetic diversity and prophage-like effect [40,41]. In *Actinobacteria*, Rothia, a commensal turned opportunist, is associated with atrophic glossitis [42] and cystic fibrosis [43]. *Pauljensenia* was linked to preeclampsia [44], and it may stimulate inflammation and immune responses by regulating the rhamnose pathway [45].

Conversely, we also observed that OSAS may reciprocally alter the oropharyngeal abundance of individual bacterial species. *Neisseria* is a Gram-negative diplococcus without spores, and is obligate aerobic. It has high nutritional requirements for growth, with an optimal pH of 7.4 to 7.6. However, OSAS, characterized by chronic intermittent hypoxia (CIH), leads to hypoxic metabolism in the body notably, possibly creating a low-oxygen and slightly acidic environment. This might be the reason why OSAS affects the abundance of *Neisseria*. Dale et al. research demonstrated that *Neisseria lactose* is a harmless symbiotic bacterium that maintains health. It colonizes the oropharyngeal upper respiratory tract and may induce the body to develop natural immunity against other pathogenic *Neisseria* species through cross-adaptive responses [46]. Therefore, the association between OSAS and *Neisseria lactosa* may have a negative cycle. *Pauljensenia* is an anaerobic microorganism that can metabolize arginine. It is commonly found in infected areas of the body. Some studies have reported that *Pauljensenia* has a colonization-promoting interaction with obesity-related bacterial species [47]. And the hypoxia and obesity associated with OSAS patients may be related to the increased abundance of *Pauljensenia*. Another Mendelian randomization analysis indicated that *Pauljensenia* is associated with a reduced incidence of bronchitis [48]. Although the association evidence between certain bacterial strains and OSAS remains uncertain, the crucial role of the oropharyngeal microbiome as the main source of the respiratory microbiota and its impact on respiratory system health has been emphasized. The interrelationships among them have affirmed the guiding influence on public health and clinical practice. The targeted, strict regulation of oropharyngeal microbiome balance holds great potential in reducing the risk of OSAS.

The existing clinical studies on the impact of OSAS treatments on oropharyngeal microecology can also illustrate the interaction between the two. The potential influence of continuous positive airway pressure (CPAP) treatment on the oropharyngeal microenvironment, including humidity, airflow, mucosal dryness, local inflammation and changes in local oxygen content, may cause the effects of bacterial colonization and proliferation. And a research from Moscow State University proved that the amount of saliva and the secretion rate decreased with the severity of OSAS [49]. The occurrence of dry mouth symptoms may lead to changes in a large number of oropharyngeal microorganisms and their metabolites, thereby disrupting and destroying oropharyngeal microecology. CPAP intervention (wearing a mouth-nose face mask supplemented with constant-temperature humidification) prevents excessive exposure of the oropharynx to the external environment and evaporation of moisture, reshaping a favorable and aerobic oropharyngeal environment, reducing the abundance of bacteria and other microorganisms that tend to grow in anaerobic environments significantly, and helping to stabilize the oropharyngeal microbial community. Ko et al. used 16S ribosomal ribonucleic acid (rRNA) sequencing and bioinformatics analysis to find that compared with those in healthy individuals, the relative abundance of Porphyromonas and Aggregatibacter in OSAS patients was higher, and the levels of pro-inflammatory cytokines were increased [50]. A whole-genome metagenomic analysis suggested that after 3 months of CPAP treatment, the oral microbiome and metabolic pathways of patients with severe OSAS could be significantly changed [51]. Individualized multimodal treatment plans for OSAS (such as upper airway surgery) have been effective in increasing the local lumen area of the airway and reducing dynamic collapse of the airway wall [52]. The surgical treatment can have an impact on the local anatomical structure of the airway [53], tissue healing, inflammatory state and subsequent microbial composition. In a study investigating the characteristics of saliva microbiome in children with OSAS, it was found that the abundance of *Prevotella* sp_HMT_396 in individuals who underwent adenoidectomy and tonsillectomy decreased sharply [54]. These intervention factors may significantly alter the structure of the oropharyngeal microbial community, which may indirectly and powerfully prove the close relationship between OSAS and microbial dysbiosis.

Consistent with prior findings, our results indicate reduced *Prevotella* and *Bacteroides* in severe OSAS [51] and increased *Streptococcus* in high-altitude hypoxia [8]. Overall, an elevated *Firmicutes*/*Bacteroidetes* (F/B) ratio is associated with an elevated risk of OSAS. The increase in the proportions of these two phyla was also observed in aging [55], obesity [56], diabetes [57], males [58] and high-altitude populations [59]. Meanwhile, OSAS is itself also closely related to the aforementioned factors such as aging and high-altitude residence [60], and OSAS is prone to be accompanied by metabolic disorders of blood glucose and lipid levels. Moreover, an increase in F/B also limits the diversity of microorganisms, representing an imbalance in the microecology within the body. Therefore, it can be reasonably associated that microecological changes have a certain influence on the occurrence of OSAS and its related complications.

These microecological changes, which are harmful to physical health, may be partly related to short-chain fatty acids (SCFAs). The F/B ratio roughly inversely correlates with SCFA production [61]. OSAS patients show reduced SCFA-producing bacteria, and acetate supplementation mitigates gut inflammation and hypertension in models [62]. SCFAs (e.g. acetate, propionate and butyrate) enhance epithelial barrier integrity, reduce mucosal inflammation and modulate enteric neural and immune responses [63]. Butyrate exerts anti-inflammatory effects via epithelial and immune cells [64], improves insulin sensitivity [65] and influences gut‒brain communication, impacts on mental state and neurological function positively [66]. It also modulates vagal activity, promotes the synthesis of neuroprotective factors and protects dopaminergic neurons [67]. Given that OSAS involves chronic inflammation, the research conducted by Huang et al. has proved that SCFAs may suppress NF-κB and oxidative stress [68]. Butyrate deficiency is linked to sarcopenia and significantly decreased richness of the intestinal microbiota [69]. And a large-sample cohort study on aging in Japan suggested that increased SCFA intake reduces the risk of muscle strength loss in elderly [70]. Specific SCFA-producing bacteria such as *Roseburia* and *Faecalibacterium* are reduced in sarcopenia [71], and the BIOSPHERE study demonstrated that *Christensenellaceae* and *Barnesiellaceae* are depleted in frailty [72]; the former produces butyrate/acetate and interacts with *Prevotella* and *Akkermansia* [73], exerting its probiotic activity to improve the structure of the microbial community. Conversely, probiotic-induced SCFA elevation improves insulin sensitivity and muscle mass in rats [74]. Therefore, it suggests SCFAs may protect against OSAS via anti-inflammatory, improvement of metabolic and neuromuscular mechanisms.

Furthermore, growing evidence links the microbiota to respiratory and neural function. Germ-free mice exhibit sleep abnormalities, and fecal transplant from insomnia patients to mice recapitulates sleep disturbances. And the mice experiment suggests that the microbial metabolite butyrate can promote sleep by regulating the activity of orexin neurons in the lateral region of the hypothalamus [75]. Notably, orexin levels are elevated in OSAS patient cerebrospinal fluid [76]; orexin neurons regulate arousal, feeding and energy balance—chronic activation/excitement promotes insomnia and obesity [77], which are tightly linked to OSAS. A recent Bayesian network meta-analysis indicated that multi-strain probiotics may be a potential option for improving BMI, reducing blood lipids and regulating leptin and high-sensitivity CRP levels [78], suggesting that probiotics can help manage weight and related metabolic issues by regulating the intestinal microbiota. It is known that probiotics can generally increase the levels of SCFAs in the intestines and circulation, thereby maintaining good health. Meanwhile, most OSAS patients are accompanied by leptin resistance [79]. Therefore, comprehensively, it is reasonable and feasible to explore the causal role of microbiota-metabolite-immune pathways in regulating disorders such as insomnia and symptoms of OSAS and related metabolism.

### Limitations

Although this study has innovatively revealed the causal relationship between the oropharyngeal microbiota and OSAS, it also has several inherent limitations. Our MR analysis used East Asian datasets, enhancing the relevance for this population but limiting generalizability. Because the incidence and phenotypic characteristics of OSAS vary across different geographical regions [80]. Moreover, craniofacial morphology, obesity prevalence, lifestyle factors, dietary habits and environmental exposures differ among populations and may affect the pathophysiology and microbiome composition of OSAS. Therefore, the occurrence and patterns of oropharyngeal microbial ecological imbalance related to OSAS proposed in this study may not be directly generalized to other geographical regions. However, these results may mainly highlight the possible causes of local throat collapse and systemic dysfunction due to oropharyngeal microbial imbalance, which has certain research guiding significance. Age-stratified microbial data were unavailable, though we discussed aging effects based on the literature. Owing to the stipulated conditions of linkage disequilibrium and the very detailed classification of bacterial strains (at the species level) before the analysis, as well as the limited number of SNPs corresponding to each strain, some bacterial IVs consisted of single SNPs, necessitating Wald ratio methods—this reduces confounding but may overlook pleiotropy. This can effectively reduce the interference of confounding factors on the association between exposure and outcome, making causal inference more reliable. However, if there are multiple genetic regulatory mechanisms, the comprehensive analysis ability of this method has been weakened and the sensitivity argument is insufficient. Moreover, while this study revealed the causal relationship between multiple bacterial species and OSAS at the species level, further FDR correction (*P*_FDR_ > 0.1) indicated that it could allow for a very low potential rate of false positives, suggesting cautious interpretation. Additionally, the statistical tools used in this study may not have completely eliminated horizontal heterogeneity. Moreover, LPR, as a potential bias, may affect the observed association between the oropharyngeal microbiota and OSAS. Although MR reduces confounding, the mechanisms related to esophageal reflux may still play a role in shaping the microbial ecosystem. Future discussions should further refine this as a possible biological mediator. Although there may be some limitations, as mentioned above, they are not sufficient to negate the significant value of the novel etiological perspective. Future studies in a multi-center and multi-ethnic and multiple statistical methods should be advocated and planned.

## Conclusions

This study implicates that disturbances in specific bacterial species in the oropharynx could cause or exacerbate OSAS. The onset and development of OSAS may be related to inflammatory responses and metabolic abnormalities such as short-chain fatty acids caused by dysbiosis of the oropharyngeal microbiota. The host-microbiome co-evolution and interaction play a vital role in health maintenance and improvement. Moving beyond previous correlative evidences, our current genetic approach further proves the causal relationship between the oropharyngeal microbiota and OSAS. This highlights the importance of correcting the disorder of the oropharyngeal microecology for improving sleep health and reducing the risk of related diseases. Therefore, detecting the oropharyngeal microbiota and applying probiotics to maintain the stability of oropharyngeal microecology may be potentially novel strategies for enriching and optimizing individualized prevention and treatment for OSAS.

## Supplementary Material

STROBE MR checklist fillable.docx

## Data Availability

This study utilized publicly available data.

## Importance

This work is crucial as it resolves a key gap: prior studies only linked oropharyngeal bacteria to OSAS (a global health issue affecting 1 billion people) but not causation. Using a robust genetic method (Mendelian randomization), it indicates a two-way causal link: imbalanced oral bacteria (e.g. more *Streptococcus* mgs 2253, fewer *Prevotella* mgs 3161 and higher *Firmicutes*/*Bacteroidetes* ratio) raises OSAS risk, while OSAS also alters specific microbial abundances. Beyond correlations, this expands OSAS's etiological understanding and offers novel prevention/treatment strategies, such as targeting oral microbiota balance.
