# Supplementary material for: Genetic predictive links between oropharyngeal microecology and obstructive sleep apnea syndrome: expanding the perspective of etiology
Source: J Oral Microbiol. 2026 Aug 18;18(1):2701568. doi: 10.1080/20002297.2026.2701568 (PMC13487851; doi:10.1080/20002297.2026.2701568)
Supplement: Supplementary file 1 [file ZJOM_A_2701568_SM6057.docx]

**STROBE-MR checklist of recommended items to address in reports of Mendelian randomization studies**^1^ ^2^

| **Item No.** | **Section** | **Checklist item** | **Page No.** | **Relevant text from manuscript** |
| --- | --- | --- | --- | --- |
| 1 | **TITLE and ABSTRACT** | Indicate Mendelian randomization (MR) as the study’s design in the title and/or the abstract if that is a main purpose of the study | 1 | Two-sample Mendelian randomization (MR) bidirectional analysis was conducted using methods such as Wald ratio and inverse-variance weighted (IVW). |
|  | **INTRODUCTION** |  |  |  |
| 2 | **Background** | Explain the scientific background and rationale for the reported study. What is the exposure? Is a potential causal relationship between exposure and outcome plausible? Justify why MR is a helpful method to address the study question | 1-2 | Mendelian randomization (MR) has emerged as a robust method in genetic epidemiology that uses genetic variants as instrumental variables (IVs) to assess whether an exposure causally influences an outcome. Oropharyngeal microecology is the exposure. |
| 3 | **Objectives** | State specific objectives clearly, including pre-specified causal hypotheses (if any). State that MR is a method that, under specific assumptions, intends to estimate causal effects | 1-2 | Objective: To explore the potential causal relationship between oropharyngeal microecology and OSAS. Most existing studies only describe associations between microbial ecology and OSAS without inferring causality. The hypothesis is that changes in the abundance of specific oropharyngeal bacterial species would cause OSAS. |
|  | **METHODS** |  |  |  |
| 4 | **Study design and data sources** | Present key elements of the study design early in the article. Consider including a table listing sources of data for all phases of the study. For each data source contributing to the analysis, describe the following: |  |  |
|  | a) | Setting: Describe the study design and the underlying population, if possible. Describe the setting, locations, and relevant dates, including periods of recruitment, exposure, follow-up, and data collection, when available. | 3 | “Data Sources” |
|  | b) | Participants: Give the eligibility criteria, and the sources and methods of selection of participants. Report the sample size, and whether any power or sample size calculations were carried out prior to the main analysis | 3 | This study applied the oropharyngeal microbiota communities of data was from China Nucleotide Sequence Archive (CNSA) (https://ftp.cngb.org/pub/CNSA/data5/CNP0001664/), and the corresponding gene sequencing data were derived from 2,017 swab samples taken from the dorsal surface of the tongue and the oropharynx for 1,915 individuals in China. The OSAS corresponding SNPs data were derived from the ebi-a-GCST90018696 dataset in the genome-wide association studies (GWAS), which included 473 cases and 177,864 controls, and the total number of SNPS was 12,454,637. |
|  | c) | Describe measurement, quality control and selection of genetic variants | 3 | The threshold for identifying relevant SNPs was set at P < 1×10−5 were retained to ensure the robustness of the IVs, which was closely related to the phenotype. Moreover, ensured that SNPs were independent of each other and filtered out data related to linkage disequilibrium (r2 = 0.001, genetic distance = 10,000). Calculated and examined the F-statistic size of each SNPs. To eliminate the influence of weak instrumental variables, retained the SNPs corresponding to F > 10 to ensure the robustness of IVs. |
|  | d) | For each exposure, outcome, and other relevant variables, describe methods of assessment and diagnostic criteria for diseases | 3 | “Data Sources” |
|  | e) | Provide details of ethics committee approval and participant informed consent, if relevant | 10 | Not applicable. No additional ethical approvals were required. Each original publication clearly states the ethical approval and informed consent for each of the studies included. All detailed studies followed the Declaration of Helsinki. |
| 5 | **Assumptions** | Explicitly state the three core IV assumptions for the main analysis (relevance, independence and exclusion restriction) as well assumptions for any additional or sensitivity analysis | 3 | the three main principles confirmed previously: (a) The correlation assumption, indicating that IVs were strongly associated with the exposure factors; (b) The independence assumption, meaning IVs should not correlate with the outcome through confounding factors; (c) The exclusive assumption, ensuring IVs influenced the outcome solely through the exposure factors without alternative pathways. |
| 6 | **Statistical methods: main analysis** | Describe statistical methods and statistics used |  |  |
|  | a) | Describe how quantitative variables were handled in the analyses (i.e., scale, units, model) | 3 | “Data Sources” |
|  | b) | Describe how genetic variants were handled in the analyses and, if applicable, how their weights were selected | 3 | The threshold for identifying relevant SNPs was set at P < 1×10−5 were retained to ensure the robustness of the IVs, which was closely related to the phenotype. Moreover, ensured that SNPs were independent of each other and filtered out data related to linkage disequilibrium (r2 = 0.001, genetic distance = 10,000). Calculated and examined the F-statistic size of each SNPs. |
|  | c) | Describe the MR estimator (e.g. two-stage least squares, Wald ratio) and related statistics. Detail the included covariates and, in case of two-sample MR, whether the same covariate set was used for adjustment in the two samples | 3 | “Design Scheme” Meanwhile, potential confounding factors such as smoking, which might affect the forward and reverse exposures in the database (P < 5×10−5), were removed. Since this was a bidirectional analysis, the selection method of IVs during the reverse analysis was the same as above. |
|  | d) | Explain how missing data were addressed | 3 | The information on microbial genetics presented in this article is all recorded in public data. “Data Sources” |
|  | e) | If applicable, indicate how multiple testing was addressed | 4 | In order to avoid false positivity in the association results due to multiple tests, false discovery rate (FDR) correction was performed, and PFDR < 0.1 was considered statistically significant. |
| 7 | **Assessment of assumptions** | Describe any methods or prior knowledge used to assess the assumptions or justify their validity | 3-4 | In the analysis, the results of the Inverse Variance weighted (IVW) method and the Wald ratio method were mainly used, and the analytical methods such as MR-Egger were adopted as supplementary methods. Furthermore, the calculation of a single SNP effect was analyzed using the Wald ratio method. |
| 8 | **Sensitivity analyses and additional analyses** | Describe any sensitivity analyses or additional analyses performed (e.g. comparison of effect estimates from different approaches, independent replication, bias analytic techniques, validation of instruments, simulations) | 4 | The MR-Egger intercept term was analyzed to determine the horizontal pleiotropy. If the Egger-intercept was infinitely close to 0 or the P value was greater than 0.05 with no statistical significance, it declared that there was no gene pleiotropy. The Cochran's Q statistic was used to evaluate heterogeneity. If P > 0.05, it indicated the absence of heterogeneity. Funnel plots were drawn to observe the distribution of SNPs, and the reliability of MR Analysis was evaluated by symmetry. Sensitivity analysis was conducted using the "Leave-one-out" method (that was, removing individual SNP seriatim and analyzing the combined effect of the remaining SNPs) to evaluate one by one whether a single SNP had an impact on the association between exposure factors and outcomes. |
| 9 | **Software and pre-registration** |  |  |  |
|  | a) | Name statistical software and package(s), including version and settings used | 3 | Using R 4.2.1 two-sample MR Software |
|  | b) | State whether the study protocol and details were pre-registered (as well as when and where) |  | Not Applicable |
|  | **RESULTS** |  |  |  |
| 10 | **Descriptive data** |  |  |  |
|  | a) | Report the numbers of individuals at each stage of included studies and reasons for exclusion. Consider use of a flow diagram | 4 | “3.1 Oropharyngeal Microbiota in MR Analysis” |
|  | b) | Report summary statistics for phenotypic exposure(s), outcome(s), and other relevant variables (e.g. means, SDs, proportions) | 4 | “3.2 Forward Associations: Oropharyngeal Microbiota on OSAS  The association analysis between oropharyngeal microbiota and OSAS was primarily conducted using the inverse-variance weighted (IVW) and Wald ratio methods. The results revealed the following:……” |
|  | c) | If the data sources include meta-analyses of previous studies, provide the assessments of heterogeneity across these studies |  | It does not include the previous meta-study. |
|  | d) | For two-sample MR:  i.  Provide justification of the similarity of the genetic variant-exposure associations between the exposure and outcome samples  ii.  Provide information on the number of individuals who overlap between the exposure and outcome studies | 2-3 and 4 | I. “…… However, most existing studies only describe associations between microbial ecology and OSAS without inferring causality.” ii. A total of 1082 oropharyngeal microbial taxa were included in this study, spanning 10 phyla, 15 classes, 30 orders, 52 families, and 199 genera. After screening based on the above criteria, 5384 SNPs associated with oropharyngeal microbiota were identified. All F-statistics exceeded 10, thereby eliminating bias related to weak instrumental variables. |
| 11 | **Main results** |  |  |  |
|  | a) | Report the associations between genetic variant and exposure, and between genetic variant and outcome, preferably on an interpretable scale | 1 | Interferences in the abundance of specific strains of oropharynx (overall, the Firmicutes/Bacteroidetes phylum ratio ↑) raised the risk of OSAS, such as increased Streptococcus mgs 2253 (OR=4.280) and decreased Prevotella mgs 3161 (OR=0.218). Conversely, OSAS also affected the abundance of some oropharyngeal microorganisms, such as a decrease in Neisseria lactamica mgs 343 and an increase in Pauljensenia mgs 2308. |
|  | b) | Report MR estimates of the relationship between exposure and outcome, and the measures of uncertainty from the MR analysis, on an interpretable scale, such as odds ratio or relative risk per SD difference | 4 and 5 | “3.2 Forward Associations: Oropharyngeal Microbiota on OSAS” ”3.4 Reverse Associations: OSAS on Oropharyngeal Microbiota” Among them, specific OR values are listed. |
|  | c) | If relevant, consider translating estimates of relative risk into absolute risk for a meaningful time period |  | Not Applicable |
|  | d) | Consider plots to visualize results (e.g. forest plot, scatterplot of associations between genetic variants and outcome versus between genetic variants and exposure) | 19-23 | Illustration of the plots of relevant results. |
| 12 | **Assessment of assumptions** |  |  |  |
|  | a) | Report the assessment of the validity of the assumptions | 4 and 5 | The corresponding data results all have their respective P-value sizes, as well as consistency in the direction of the combined effect value β obtained from the different analytical methods. |
|  | b) | Report any additional statistics (e.g., assessments of heterogeneity across genetic variants, such as *I^2^*, Q statistic or E-value) | 4 and 5 | “3.3 Sensitivity Analysis of Forward Associations” ”3.5 Sensitivity Analysis of Reverse Associations” |
| 13 | **Sensitivity analyses and additional analyses** |  |  |  |
|  | a) | Report any sensitivity analyses to assess the robustness of the main results to violations of the assumptions | 4 and 5 | ……for Streptococcus mgs 2821 (βMR-Egger = 4.515, βIVW = -1.074) and Gemella mgs 2318 (βMR-Egger = 3.363, βIVW = -0.870), the comprehensive effect values β directions obtained through MR-Egger and IVW analyses were inconsistent, suggesting instability in the causal relationship. Therefore, these two bacteria were excluded from subsequent discussions. “3.3 Sensitivity Analysis of Forward Associations” ”3.5 Sensitivity Analysis of Reverse Associations” |
|  | b) | Report results from other sensitivity analyses or additional analyses | 4 and 5 | “3.3 Sensitivity Analysis of Forward Associations” ”3.5 Sensitivity Analysis of Reverse Associations” |
|  | c) | Report any assessment of direction of causal relationship (e.g., bidirectional MR) | 4 and 5 | “3.2 Forward Associations: Oropharyngeal Microbiota on OSAS” ”3.4 Reverse Associations: OSAS on Oropharyngeal Microbiota” |
|  | d) | When relevant, report and compare with estimates from non-MR analyses | 2 and 6 | In the Introduction section, we presented the previous studies which described the correlation between OSAS and the oral pharyngeal microecology. Moreover, the oropharynx and gut represent the body’s largest microbial reservoirs, interconnected via the oral-gut microbiome axis [21]. Oropharyngeal microbes can translocate to the gut via hematogenous routes or through saliva swallowing. Under the mutual influence and joint effect between these two planting locations of microorganisms, they can regulate the host's immunity or induce diseases. For example, the research from Xiyuan Hospital, China Academy of Chinese Medical Sciences, found that elevated Fusobacterium nucleatum in the oropharynx correlates with oral-gut dysbiosis characterized by increased Lactobacillus co-colonization, which is associated with the aggravation of myocardial ischemia-reperfusion injury in diabetes [22]. Thus, oropharyngeal ecological imbalance may contribute to systemic dysfunction. |
|  | e) | Consider additional plots to visualize results (e.g., leave-one-out analyses) | 22-23 | Bidirectional of leave-one-out analyses plots are present. |
|  | **DISCUSSION** |  |  |  |
| 14 | **Key results** | Summarize key results with reference to study objectives | 1 and 10 | This study emphasizes the causal relationship between oropharyngeal microecology and OSAS, expands the new etiological mechanism, and provides potential novel strategies for the prevention and treatment of OSAS. This study implicates that disturbances in specific bacterial species in the oropharynx can cause or exacerbate OSAS. Moving beyond previous correlative evidences, our current genetic approach further proves the causal relationship between the oropharyngeal microbiota and OSAS. |
| 15 | **Limitations** | Discuss limitations of the study, taking into account the validity of the IV assumptions, other sources of potential bias, and imprecision. Discuss both direction and magnitude of any potential bias and any efforts to address them | 9-10 | “Limitations” |
| 16 | **Interpretation** |  |  |  |
|  | a) | Meaning: Give a cautious overall interpretation of results in the context of their limitations and in comparison with other studies | 6-10 | “Discussion” This study implicates that disturbances in specific bacterial species in the oropharynx could cause or exacerbate OSAS. |
|  | b) | Mechanism: Discuss underlying biological mechanisms that could drive a potential causal relationship between the investigated exposure and the outcome, and whether the gene-environment equivalence assumption is reasonable. Use causal language carefully, clarifying that IV estimates may provide causal effects only under certain assumptions | 6-9 | In the second to seventh paragraphs of the Discussion section, examples of other related studies that support our current results are presented, along with the possible mechanisms involved in the causal relationship. |
|  | c) | Clinical relevance: Discuss whether the results have clinical or public policy relevance, and to what extent they inform effect sizes of possible interventions | 10 | It highlights the importance of correcting the disorder of the oropharyngeal microecology for improving sleep health and reducing the risk of related diseases. Therefore, detecting the oropharyngeal microbiota and applying probiotics to maintain the stability of the oropharyngeal microecology may be potentially novel strategies for enriching and optimizing individualized prevention and treatment for OSAS. |
| 17 | **Generalizability** | Discuss the generalizability of the study results (a) to other populations, (b) across other exposure periods/timings, and (c) across other levels of exposure | 9-10 | Our MR analysis used East Asian datasets, enhancing relevance for this population but limiting generalizability. Age-stratified microbial data were unavailable, though we discussed aging effects based on literature. Due to the stipulated conditions of linkage disequilibrium and the very detailed classification of bacterial strains (at the species level) before the analysis, as well as the limited number of SNPs corresponding to each strain, some bacterial IVs consisted of single SNPs, necessitating Wald ratio methods — this reduces confounding but may overlook pleiotropy.…… |
|  | **OTHER INFORMATION** |  |  |  |
| 18 | **Funding** | Describe sources of funding and the role of funders in the present study and, if applicable, sources of funding for the databases and original study or studies on which the present study is based | 10 | This work was supported by grants 82270345 from the National Natural Science Foundation of China, YGLX202324 from Beijing Municipal Healthcare Administration and 2024-1-2061 from First-Launch Project of Beijing Municipal Health Commission. The funders had no direct role in the design, data collection, analysis, interpretation, or writing of the manuscript. |
| 19 | **Data and data sharing** | Provide the data used to perform all analyses or report where and how the data can be accessed, and reference these sources in the article. Provide the statistical code needed to reproduce the results in the article, or report whether the code is publicly accessible and if so, where | 3 and 10 | This study utilized publicly available data. |
| 20 | **Conflicts of Interest** | All authors should declare all potential conflicts of interest | 11 | The authors declared no competing interests for this work. |

This checklist is copyrighted by the Equator Network under the Creative Commons Attribution 3.0 Unported (CC BY 3.0) license.

1.	Skrivankova VW, Richmond RC, Woolf BAR, Yarmolinsky J, Davies NM, Swanson SA, et al. Strengthening the Reporting of Observational Studies in Epidemiology using Mendelian Randomization (STROBE-MR) Statement. JAMA. 2021;under review.

2.	Skrivankova VW, Richmond RC, Woolf BAR, Davies NM, Swanson SA, VanderWeele TJ, et al. Strengthening the Reporting of Observational Studies in Epidemiology using Mendelian Randomisation (STROBE-MR): Explanation and Elaboration. BMJ. 2021;375:n2233.
